# Characterization of Heavy Metal Ions Removal from Water by Improved Cuttlebone Powder with Magnetic Fe_3_O_4_ Nanoparticle as a Bioadsorbent

**DOI:** 10.1002/gch2.202400107

**Published:** 2025-01-07

**Authors:** Aida Didaran, Mahnaz Sadat Sadeghi, Parisa Nejatkhah Manavi, Mohammad Rabbani

**Affiliations:** ^1^ Department of Marine Biology North Tehran Branch, Islamic Azad University Tehran Iran; ^2^ Department of Marine Chemistry North Tehran Branch, Islamic Azad University Tehran Iran

**Keywords:** adsorption, heavy metals, *Sepia pharaonic*, water treatment

## Abstract

Scientists are constantly striving to develop improved methods for reducing or eliminating the discharge of harmful heavy metals into drinking water sources and the environment. In light of this, this study investigates the potential of utilizing a naturally available and sustainable bio‐waste material, *Sepia pharaonis* cuttlebone Powder (SCP), as an exceptionally effective adsorbent for the adsorption of Ni (II), Pb (II), Cu (II), and Fe (II) ions. SCP is also characterized by Fourier‐transform infrared Spectroscopy (FTIR), Scanning Electron Microscopy (SEM), Xray diffraction (XRD), and Energy dispersive Xray (EDX) analysis. The results reveal that the highest absorption values for Ni (II), Cu (II), and Fe (II) ions are observed at pH 8, while Pb (II) ions exhibit the highest absorption at pH 4. The absorption percentage of the ions displays an increasing trend with the amount of SCP used, as well as with contact time and magnetizing SCP duration. Additionally, the addition of Fe_3_O_4_ to SCP significantly improves the absorption rate of the samples. Both Langmuir and Freundlich adsorption models are found to fit the experimental data well. Overall, SCP, with or without Fe_3_O_4_ nanoparticles, shows great promise for water remediation processes by effectively removing heavy metals.

## Graphical abstract

This study investigates the potential of utilizing a naturally available and sustainable bio‐waste material, *Sepia pharaonis* cuttlebone Powder (SCP), as an exceptionally effective adsorbent for the adsorption of Ni (II), Pb (II), Cu (II), and Fe (II) ions. It is found that SCP when combined with Fe_3_O_4_ nanoparticles and magnetized, shows excellent adsorption efficiency for these metal ions.

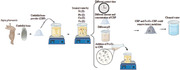

## 1 Introduction

Water contamination by heavy metals has become a significant environmental concern, posing serious risks to human health and ecosystems.^[^ ^1^, ^2^ ^]^ Heavy metals, such as Ni (II), Pb (II), Cu (II), and Fe (II), are persistent pollutants that can accumulate in water sources through industrial activities, mining, agricultural runoff, and improper disposal of waste.^[^ ^3^, ^4^ ^]^ These metals are known to be toxic, even at low concentrations, and can cause various health issues, including organ damage, neurological disorders, and carcinogenic effects.^[^ ^5^, ^6^ ^]^ Traditional methods for heavy metal removal, such as chemical precipitation and ion exchange, have proven to be effective but are often associated with several drawbacks.^[^ ^1^, ^7^ ^]^ These methods require the use of expensive chemicals, large amounts of energy, and complex processes.^[^ ^1^, ^8^, ^9^ ^]^ Furthermore, they may generate harmful byproducts that require proper disposal, adding to the overall environmental impact.^[^ ^3^, ^10^ ^]^ Therefore, there is a growing demand for sustainable and cost‐effective alternatives that can efficiently eliminate heavy metals from water and wastewater.^[^ ^1^, ^10^, ^11^, ^12^ ^]^ Researchers are exploring natural adsorbents derived from various sources, as they offer several advantages over conventional methods.^[^ ^13^, ^14^ ^]^ Natural adsorbents are abundant, renewable, and often exhibit high adsorption capacities and selectivity toward heavy metals.^[^ ^13^, ^15^, ^16^ ^]^ Among these natural adsorbents, *Sepia pharaonis* (Cuttlefish) cuttlebone powder (SCP) has gained attention due to its unique properties and potential applications in heavy metal removal.^[^ ^4^, ^17^ ^]^ Sepia belongs to the cephalopod family and is classified within the order of octopuses and cephalopods.^[^ ^18^ ^]^ Sepia is widely consumed as a popular seafood worldwide, and China currently holds the position of being the largest exporter of squid globally.^[^ ^19^ ^]^ In 2018, the export of squid reached ≈250 000 tons, representing a growth of 2.3% compared to the previous year.^[^ ^20^ ^]^ The internal skeleton of sepia, aside from being naturally found in the waters where this animal resides, is also a valuable byproduct of the marine food industry.^[^ ^21^, ^22^ ^]^ The primary component of the sepia endoskeleton, constituting over 60% of its composition, is calcium carbonate in the form of aragonite crystals.^[^ ^21^, ^22^ ^]^ Recent studies have demonstrated the remarkable ability of sepia's internal bone to efficiently adsorb copper, cadmium, lead, chromium, and fluoride cations, further highlighting its potential for heavy metal removal.^[^ ^1^, ^18^, ^23^ ^]^

Also, there has been a growing interest in using Fe_3_O_4_ nanoparticles with magnetic properties to remove heavy metal ions from water.^[^ ^24^ ^]^ These nanoparticles can be easily separated from water using a magnetic field.^[^ ^25^ ^]^ Due to their large surface area and ability to be modified, they can efficiently adsorb heavy metal ions like lead, cadmium, and mercury from polluted water sources.^[^ ^26^ ^]^ The size and surface chemistry of the nanoparticles can be controlled during synthesis, making it possible to customize their adsorption properties.^[^ ^24^, ^25^ ^]^

Therefore, this study introduces a novel approach for heavy metal removal by combining SCP with magnetic Fe_3_O_4_ nanoparticles, creating a hybrid bioadsorbent with enhanced adsorption capacity and ease of recovery. The research systematically investigates the influence of key adsorption parameters on the removal of Ni (II), Pb (II), Cu (II), and Fe (II) ions, providing a comprehensive understanding of the adsorption process and its efficiency. Both Langmuir and Freundlich isotherm models are employed to describe the adsorption mechanism, further strengthening the potential application of SCP in water remediation. By utilizing a naturally available and sustainable bio‐waste material, this study promotes a green and environmentally friendly approach to water treatment, reducing reliance on synthetic materials.

## 2 Results and Discussion

### 2.1 Characterization of the SCP

#### 2.1.1 FTIR Spectroscopy

**Figure 1:**
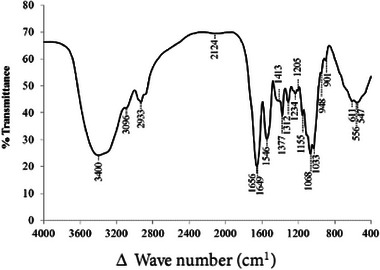
Fourier transform infrared (FT‐IR) spectrum image of SCP.

It has been found that metal removal by biomass materials from aqueous solutions depends largely on the functional groups on the surface of the biomass.^[^ ^27^ ^]^ We therefore analyzed the changes of functional groups of SCP and the result is depicted in **Figure** **1**. The peaks observed at 556, 1068, and 1649 cm^−1^ correspond to the functional group C–O–C.^[^ ^13^ ^]^ The absorption bands at 1033, 1068, and 2522 cm^−1^ indicate the existence of aragonite carbonates.^[^ ^1^ ^]^ The band observed at 1792 cm^−1^ is associated with the CO and the peak at 2933 cm^−1^ is indicative of the CH═CH group.^[^ ^1^ ^]^ Additionally, the peak at 3400 cm^−1^ signifies the presence of the –OH group.^[^ ^1^, ^2^ ^]^ Moreover, the broad absorption band of –OH and –NH at 3400 cm^−1^, and reference bands of chitin at 2933 and 3096 cm^−1^ may correspond to the chitinous constituent of SCP.^[^ ^4^ ^]^ The vibrational modes observed in the FTIR spectrum included a peak at 1155 cm⁻¹ attributed to the antisymmetric stretching of C–O–C bridges, a peak at 1377 cm⁻¹ assigned to CH₃ groups, and a peak at 1546 cm⁻¹ characteristic of amide and primary amine groups.^[^ ^8^, ^28^ ^]^

#### 2.1.2 XRD

**Figure 2:**
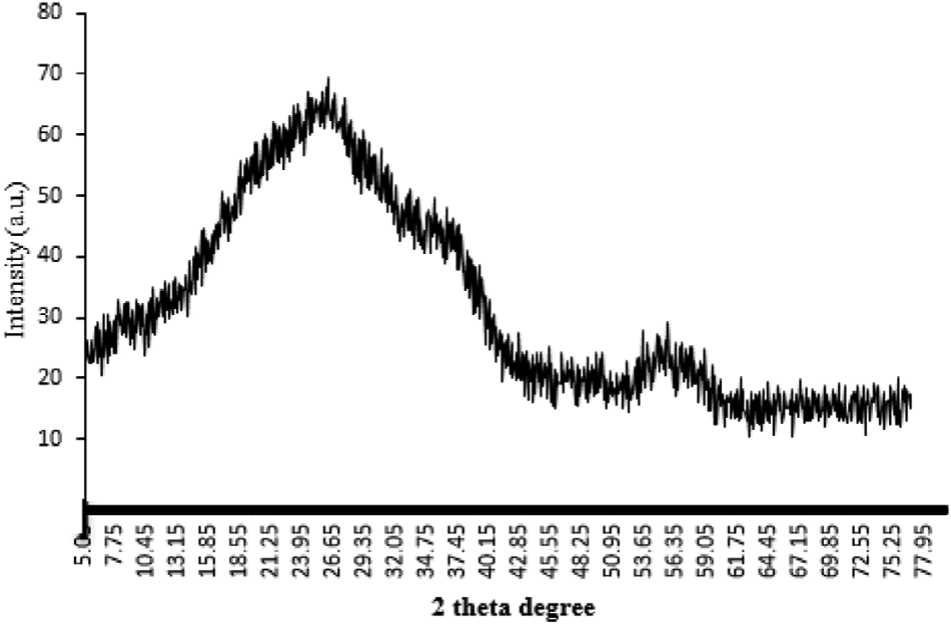
XRD graph of the SCP sample.

The XRD results for the SCP sample, shown in **Figure** **2**, reveal a broad peak at ≈27° 2θ. This peak indicates the crystalline structure of the SCP sample, consistent with the findings reported by Yazid et al.^[^ ^29^ ^]^ They reported that this spectrum indicates the presence of aragonite. These findings also align with those of Nouj et al.,^[^ ^21^ ^]^ who reported a peak at ≈30° in cuttlefish bone, confirming its crystalline structure and identifying aragonite as the sole mineral phase. Here, the most prominent peak is also located ≈30°, attributable to aragonite, while other peaks represent variations in the calcium carbonate composition. Furthermore, a broad peak between 5° and 30° 2θ is assigned to the crystalline structure of β‐chitin, the most abundant organic component of Sepia officinalis bone.^[^ ^30^ ^]^

#### 2.1.3 SEM

**Figure 3:**
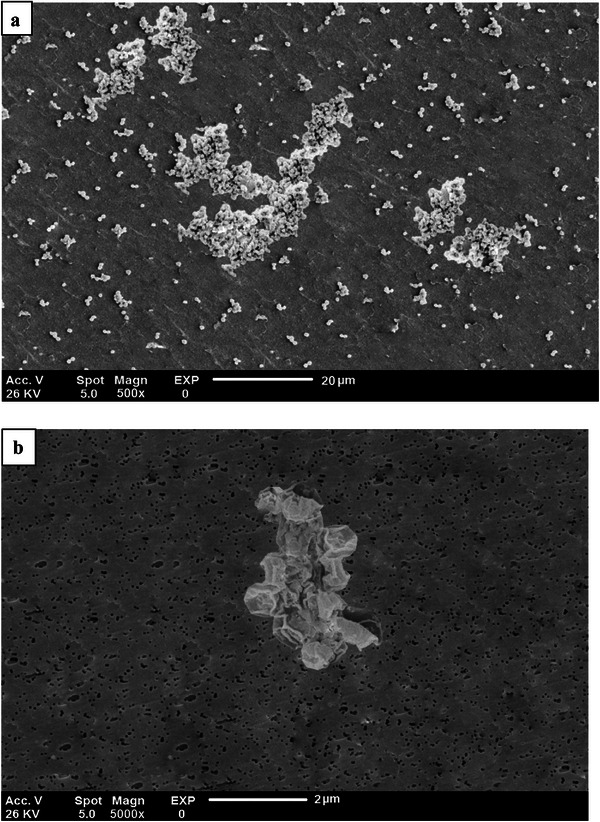
Scanning electron microscopic (SEM) images of the SCP. a) Micrograph at 500×, b) micrograph at 5000×.

SEM images of the SCP sample at magnifications of 500× and 5000× are shown in **Figure** **3**. As can be seen, the SCP powder exhibits a rough and heterogeneous structure. The presence of pores indicates a relatively porous structure, consistent with the porous nature of marine biogenic materials. These observations closely resemble the images of cuttlefish bone (Sepia) presented by Yazid et al.^[^ ^29^ ^]^ The relatively rough structure observed in Figure 3B may be attributed to the presence of chitin. It has been reported that this non‐uniform and heterogeneous structure can significantly improve the performance of these materials, enhancing their adsorption capacity.^[^ ^31^, ^32^ ^]^

#### 2.1.4 EDX

**Figure 4:**
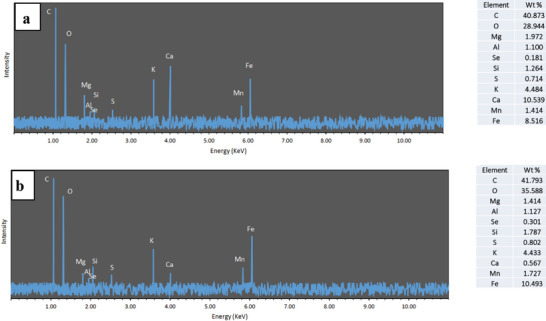
EDX spectrograms of a) SCP and b) SCP‐Fe_3_O_4_.

EDX spectroscopy (**Figure** **4**a,b) was employed to determine the elemental composition of the SCP and its modified form, SCP‐Fe_3_O_4_. Analysis of the SCP sample revealed a composition primarily consisting of carbon (40.87%), oxygen (28.94%), calcium (10.53%), and iron (8.51%). Following the addition of Fe_3_O_4_, the elemental composition shifted to 41.79% carbon, 35.58% oxygen, 0.56% calcium, and 10.49% iron. The addition of Fe_3_O_4_ clearly resulted in a significant increase in iron and oxygen content, while the calcium content decreased substantially. Minor amounts of magnesium, aluminum, silicon, and potassium were also detected in both SCP and SCP‐Fe_3_O_4_ samples. The high carbon, oxygen, and calcium content in SCP are consistent with its known composition, which is rich in CaCO_3_, along with minor amounts of protein (3–7%) and chitin (3–4%).^[^ ^33^ ^]^ The observed changes in elemental composition after chemical modification of the cuttlebone powder may be attributed to residual chemicals remaining within the sample's pores.^[^ ^1^ ^]^ These results are consistent with previous studies Nouj et al.^[^ ^21^ ^]^ and Bhagyaraj et al.^[^ ^1^ ^]^

### 2.2 Effect of Initial pH on Metal Removal of SCP

**Figure 5:**
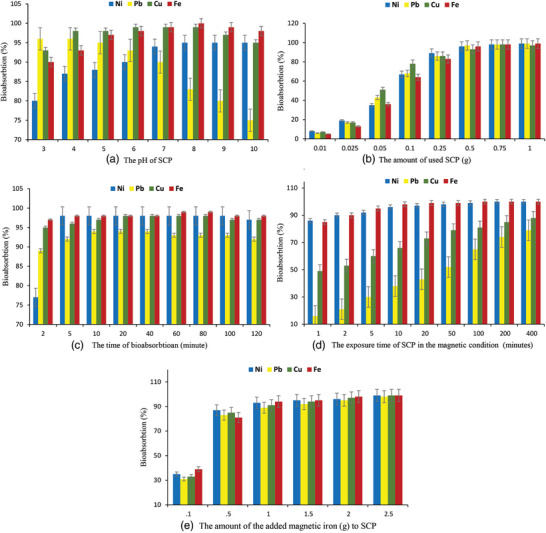
a) The results of the initial pH of SCP on biosorption of Ni (II), Pb (II), Cu (II), and Fe (II) ions. b) The results of the effect of the amount of SCP on biosorption of Ni (II), Pb (II), Cu (II), and Fe (II) ions. c) The results of the contact time of metal removal of Ni (II), Pb (II), Cu (II), and Fe (II) ions on SCP. d) The results of the exposure time of SCP in the magnetic condition on their metal removal including Ni (II), Pb (II), Cu (II), and Fe (II) ions. e) The result of adding the different amounts of magnetic iron to SCP on the absorption percentage of heavy metals including Ni (II), Pb (II), Cu (II), and Fe (II) ions.

The initial pH of the solution plays a crucial role in the adsorption process. pH is a significant factor that affects the surface charge of the sorbent, consequently influencing the amount of metal sorption.^[^ ^23^, ^31^ ^]^ The impact of pH on metal removal is attributed to the chemistry of the metal, the ionization state of the functional groups, and the ion exchangeability of the adsorbent.^[^ ^4^ ^]^ To assess the effect of this parameter, experiments were conducted within a pH range of 3–10. As shown in **Figure** **5**a, the cation removal was greatly influenced by the solution pH, and all tested metal ions were effectively adsorbed onto SCP, which contradicts the findings reported by Rahbar et al.^[^ ^4^ ^]^ The results varied for each investigated cation at different pH levels. Notably, an increasing trend was observed for Ni (II), Cu (II), and Fe (II) cations as the pH increased from 3 to 10, which in parallel with reported results on the rapid removal of cobalt (II) from aqueous solution using cuttlefish bones by Sandesh et al.^[^ ^23^ ^]^ However, Pb exhibited a reverse trend, with its adsorption rate on SCP sharply decreasing with increasing pH. One possible explanation for this outcome is that SCP mainly consists of calcium carbonate, which could result in a negative surface charge at lower pH values.^[^ ^4^, ^23^ ^]^ Consequently, when the bio‐sorbent is mixed with the sample solution, the pH is expected to increase. Also, the examined ions have the ability to easily replace and substitute calcium ions due to their capacity to form more robust insoluble carbonate salts compared to calcium ions.^[^ ^34^ ^]^ In addition, an additional mechanism for the chemisorption of the examined cations could involve micro‐precipitation and the condensation of metal hydroxides onto the bio‐sorbent.^[^ ^4^ ^]^ However, the role of surface complex formation and electrostatic adsorption mechanisms in removing metal ions from the sample solution can be attributed to the functional groups present in the chitinous material of SCP. These functional groups, including hydroxyl groups and amide groups that can be converted to amine groups in an alkaline environment, may have a relatively lesser impact on the overall adsorption process.^[^ ^35^ ^]^ In a study by Lapo et al.,^[^ ^36^ ^]^ it was found that the bioadsorbent made from the ChiFer (III) composite is less stable at pH levels below 3.0, due to the hydrolysis of chitosan and the resulting dissolution of both organic and inorganic materials in the beads. Conversely, at pH levels above 6, insoluble hydroxide precipitates form. The optimal pH range identified was between 4.5 and 5.0. In a separate study, the minimum lead adsorption efficiency for the brown algae *Nizimuddinia zanardini* was recorded at a pH of 3, achieving a value of 28.9%. In contrast, the green algae *Ulva rigida* exhibited a lower efficiency of 23.9% at the same pH level. The findings demonstrated that as the pH increased, the lead adsorption capacity of both algae showed a positive correlation, indicating an upward trend in their ability to adsorb lead.

### 2.3 Effect of the Amount of SCP on Metal Removal

To determine the optimal amount of SCP required for maximum adsorption of the mentioned metal ions in a 50 mL solution (containing 20 mg L^−1^ of cations) at an initial pH of 7, a range of 0.01–1.00 g of the adsorbent was tested. As shown in Figure 5b, the highest removal percentage (99%) for Ni (II), Pb (II), and Fe (II) cations was achieved when the amount of SCP used was 1.00 g. However, for Cu (II), the maximum removal percentage was achieved at 0.75 g of SCP. This can be attributed to effective chemisorption through the aforementioned mechanisms and the presence of numerous adsorption sites on the adsorbent at higher dosages. Consequently, increasing the amount of SCP leads to an increase in the removal percentage of the metal ions. Forutan et al.^[^ ^11^ ^]^ found that increasing the dosage of the adsorbent enhanced the absorption efficiency of lead ion concentration.

### 2.4 Effect of the Contact Time on Metal Removal

The effect of contact time on the sorption process was investigated by mixing a 20 mg L^−1^ solution of metal ions with 0.5 g of SCP for varying time intervals ranging from 2 min to 2 h (Figure 5c). The results demonstrated that SCP has the potential to remove over 90% of all the evaluated ions within just 10 min of contact time. These rapid adsorption rates indicate a strong attraction between the metal ions and SCP, possibly through chemical binding or electrostatic attraction.^[^ ^4^, ^5^ ^]^ Additionally, these results can be attributed to the electronegativity order of the metal ions, with Pb (II) (2.33), Cu (II) (1.95), and Cd (II) (1.69).^[^ ^4^ ^]^ The most likely mechanism for metal ion adsorption onto SCP is suspected to be ion exchange between Ca (II) and the tested metal ions.^[^ ^37^ ^]^ Therefore, the existence of competition between the examined metal ions and Ca (II) is reasonable, with a higher rate of exchange observed when the metal ion is more electronegative.^[^ ^35^ ^]^ Rahman et al.^[^ ^2^ ^]^ demonstrated that the bio‐adsorption patterns for Cr^6+^ and As^5+^ remained consistent across all time intervals from 30 to 180 min. In contrast, nickel exhibited a slow initial adsorption rate, which increased progressively by the 180‐min mark. Cobalt absorption, however, showed a distinct trend: it was significantly higher during the first 30 min, followed by a slight decline up to 120 min, before gradually rising again by the 180‐min mark. These findings align closely with our own results.

### 2.5 Effect of the Exposure Time of SCP in the Magnetic Condition on their Metal Removal

Figure 5d illustrates the impact of exposure time in a magnetic condition on the metal removal efficiency of SCP. The results clearly demonstrate that the highest levels of bioadsorption were achieved for iron, nickel, copper, and lead cations, with percentages of 100%, 100%, 88%, and 79% respectively. These remarkable percentages were attained during different exposure times of SCP under magnetic conditions, specifically 100, 200, 400, and 400 min for iron, nickel, copper, and lead cations respectively. Currently, magnetic fields are employed to enhance the bioadsorption capabilities of heavy metals using compounds derived from crustacean shells, like chitin and chitosan. Chitosan, in particular, serves as a prime example, as it demonstrates remarkable proficiency in chelating metals due to the presence of amine and hydroxyl groups within its molecular composition. Furthermore, its magnetic properties facilitate effortless separation from the sorption system by simply applying a magnetic field.^[^ ^38^, ^39^ ^]^

### 2.6 The Effect of Adding the Amount of Magnetic Iron to SCP on the Adsorption Percentage of Heavy Metals Lead, Nickel, Iron and Copper

As shown in Figure 5e, the inclusion of magnetized iron significantly enhanced the bioadsorption of the investigated heavy metal ions. It was observed that the adsorption rate increased noticeably when the amount of magnetic iron added to SCP was changed from 0.1 to 0.5 g. Furthermore, this trend continued as the amount of adsorption increased, with the highest adsorption rate being achieved when 2.5 g of magnetized iron was added to SCP. Many studies have mentioned that cuttlefish bone is an effective adsorbent for heavy metal ions due to its biodegradability, availability, and low cost.^[^ ^23^, ^40^ ^]^ However, it could have some drawbacks such as poor physical and chemical properties like low mechanical strength and high solubility in acid solution due to having chitin and chitosan.^[^ ^3^, ^5^, ^40^ ^]^ To address these issues, various modifications have been attempted.^[^ ^3^, ^5^, ^40^ ^]^ For instance, Zhou et al.^[^ ^41^ ^]^ discovered that crosslinking could enhance the stability of chitosan in an acid solution. Additionally, it has been found that magnetic chitosan can be easily separated from water using a permanent magnet.^[^ ^13^ ^]^ The same results as our results were obtained by Rahmi and Lelifajri^[^ ^3^ ^]^ for mercury removal by magnetic chitosan, in which the addition of Fe_3_O_4_ to chitosan could greatly improve the removal activity of mercury.

### 2.7 Adsorption Isotherms

**Figure 6:**
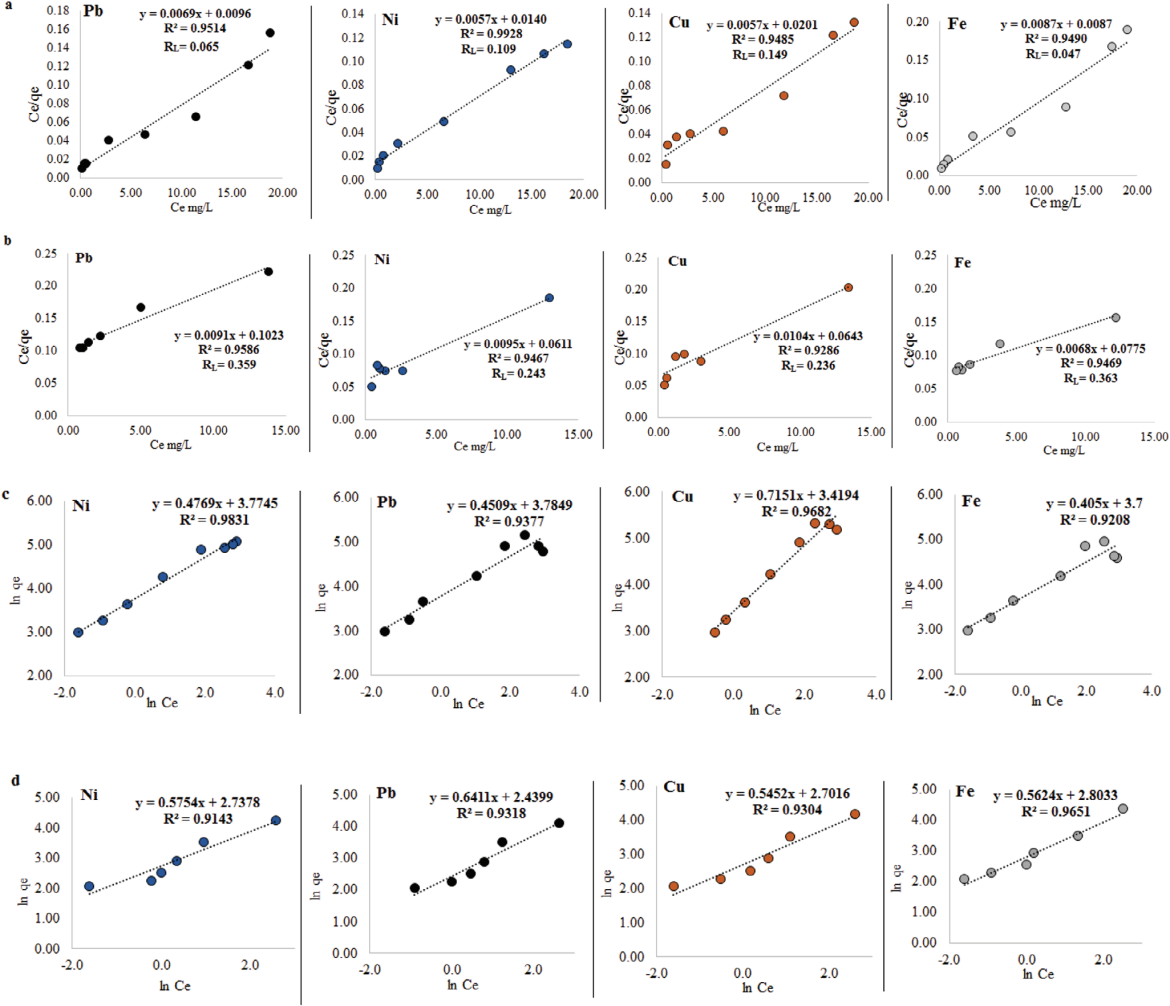
Langmuir isotherm model for Ni (II), Pb (II), Cu (II), and Fe (II) ions adsorption by different amounts of a) SCP and b) Fe_3_O_4_‐SCP, and Freundlich isotherm model for Ni (II), Pb (II), Cu (II), and Fe (II) ions adsorption by different amounts c) SCP and d) Fe_3_O_4_‐SCP is different.

Understanding the adsorption isotherms is crucial for investigating the interaction between ions and an adsorbent.^[^ ^5^ ^]^ These isotherms describe the distribution of the adsorbate between the liquid and solid phases at equilibrium.^[^ ^5^, ^23^ ^]^ They provide information about the equilibrium relationship between the amount of metal ions adsorbed by the adsorbent.^[^ ^3^ ^]^ The Langmuir adsorption isotherm model assumes monolayer adsorption, while the Freundlich adsorption isotherm model assumes multilayer adsorption.^[^ ^3^, ^40^ ^]^ To examine the adsorption isotherm of magnetic chitosan for Ni (II), Pb (II), Cu (II), and Fe (II) ions using SCP and Fe_3_O_4_‐SCP, various adsorption experiments were performed with varying amounts of SCP and Fe_3_O_4_‐SCP. The results can be seen in **Figure** **6**. Based on the R^2^ values, the most suitable adsorption isotherm models for Ni (II), Pb (II), Cu (II), and Fe (II) ions by SCP and Fe_3_O_4_‐SCP were determined. As shown in **Table** **1**, both models had R^2^ values greater than 0.9 for all mentioned ions, indicating a good fit for the adsorption isotherm models of these ions by SCP and Fe_3_O_4_‐SCP.^[^ ^40^ ^]^ According to Table 1, the Langmuir adsorption isotherm model showed that the maximum adsorption capacity (Q_m_) of Ni (II), Pb (II), Cu (II), and Fe (II) ions by SCP in this study were 175.43, 144.92, 175.43, and 114.94 mg g^−1^, respectively. Conversely, the Q_m_ values for these ions absorbed by Fe_3_O_4_‐SCP were 105.26, 109.89, 96.15, and 147.05 mg g^−1^, respectively. Furthermore, the results indicated that the R^2^ values for all adsorbed ions by SCP were lower compared to those obtained by Fe_3_O_4_‐SCP. Among the adsorbed ions, Ni (II) exhibited the highest R^2^ value in the Langmuir adsorption isotherm model when adsorbed by SCP, while the lowest R^2^ value was observed for the adsorption of Cu (II) by Fe_3_O_4_‐SCP. The separation factor/equilibrium parameter (RL), obtained from the Langmuir adsorption isotherm model, is a dimensionless parameter used to assess the favorability of the adsorption process.^[^ ^5^, ^40^ ^]^ When the RL value falls between 0 and 1, it indicates a favorable adsorption process.^[^ ^5^, ^40^ ^]^ In this study, the RL values for the sorption of Ni (II), Pb (II), Cu (II), and Fe (II) ions onto SCP and Fe_3_O_4_‐SCP were found to be within the range of 0 to 1. This suggests that the sorption of these ions on both SCP and Fe_3_O_4_‐SCP was favorable. An interesting observation is that the RL (separation factor) in all samples of ions absorbed by Fe_3_O_4_‐SCP was higher compared to SCP. Furthermore, the highest value of this index was observed in the adsorption of Fe (II) by Fe_3_O_4_‐SCP.

**Table 1:** Adsorption isotherm constants of the Langmuir and Freundlich models for Ni (II), Pb (II), Cu (II), and Fe (II) ions adsorption by different amounts c) SCP and d) Fe_3_O_4_‐SCP.

	Langmuir model				Freundlich model		
		Q_m_ [mg g^−1^]	K_L_ [L mg^−1^]	R^2^	n	K_F_ [mg g^−1^]	R^2^
SCP	Pb	144.9275	0.71875	0.9514	2.217787	44.03127	0.9377
	Ni	175.4386	0.407143	0.9928	2.096876	43.57572	0.9831
	Cu	175.4386	0.283582	0.9480	1.398406	30.55108	0.9682
	Fe	114.9425	1	0.9490	2.469136	40.44730	0.9208
Fe_3_O_4_‐SCP	Pb	109.8901	0.088954	0.9586	1.559819	11.47189	0.9318
	Ni	105.2632	0.155483	0.9467	1.737921	15.45295	0.9143
	Cu	96.15385	0.161742	0.9286	1.834189	14.90356	0.9304
	Fe	147.0588	0.087742	0.9469	1.778094	16.49900	0.9651

The adsorbent's relative adsorption capacity can be assessed by examining the value of Kf, which represents the Freundlich constant.^[^ ^5^, ^13^ ^]^ Kf predicts the quantity of metal ions per gram of sorbent at a unit equilibrium concentration.^[^ ^13^ ^]^ The parameter “n” indicates the strength and nature of the adsorption process, as well as the distribution of active sites.^[^ ^13^ ^]^ If n is less than 1, bond energies increase with surface density, whereas if n is greater than 1, bond energies decrease with the equivalent.^[^ ^3^, ^5^, ^13^ ^]^ For the adsorption of Ni (II), Pb (II), Cu (II), and Fe (II) ions by SCP, the values of Kf and n were determined to be 43.575, 44.031, 30.551, 40.447 mg g^−1^ and 2.096, 2.217, 1.398, and 2.496, respectively. In contrast, for adsorption by Fe_3_O_4_‐SCP, these values were found to be 15.452, 11.471, 14.903, 16.499 mg g^−1,^ and 1.737, 1.559, 1.834, and 1.778, respectively. It is worth noting that when Fe_3_O_4_‐SCP was used instead of SCP, the values of n and Kf decreased for the adsorption of Ni (II), Pb (II), Cu (II), and Fe (II) ions. According to the Freundlich model, n values between 1 and 10 indicate a favorable adsorption process. In this study, all the n values obtained for different amounts of SCP and Fe_3_O_4_‐SCP fell within the range of 1–10. This suggests that the sorption of Ni (II), Pb (II), Cu (II), and Fe (II) ions by the novel material was favorable.

When comparing the results of the Langmuir and Freundlich models, it is evident that different outcomes are obtained for each ion in terms of fit. For instance, when Ni (II), Pb (II), and Fe (II) ions are absorbed by SCP, both treatments exhibit the highest R^2^ values in the Langmuir model. This suggests that the Langmuir model provides a better description of the adsorption process for those ions. Therefore, this finding implies that the surface sites responsible for metal adsorption are evenly distributed.^[^ ^39^, ^42^ ^]^ This aligns with previous research conducted by Peralta et al.,^[^ ^39^ ^]^ which demonstrated the effective removal of heavy metals from water using magnetic chitosan‐based composites and described the adsorption isotherms using the Langmuir model. A stronger correlation with the Langmuir model indicates that the adsorption process is more favorable for monolayer coverage, and there is minimal interaction among the adsorbed metal ions.^[^ ^5^ ^]^ An interesting finding by Taguba et al.^[^ ^5^ ^]^ supports this observation. They discovered that the Langmuir model exhibited higher coefficients of determination (R^2^) compared to the Freundlich model when describing the adsorption of Cu (II) and Pb (II) on MnFe_2_O_4_/Chitosan Nanoadsorbents. This indicates that the Langmuir model was better suited for explaining the adsorption behavior of these metal ions on the nanoadsorbents in their study.^[^ ^5^ ^]^ On the other hand, in the adsorption of copper and iron ions by Fe_3_O_4_‐SCP, the Freundlich model yields a higher R^2^ value compared to the Langmuir model. This indicates that the Freundlich model is more suitable for describing the adsorption of copper and iron ions in this particular case.

## 3 Conclusion

This study demonstrates the potential of *Sepia pharaonis* Endoskeleton (SCP), a biomass waste, for efficient wastewater treatment by removing Ni (II), Pb (II), Cu (II), and Fe (II) ions. Batch experiments revealed that the adsorption process is influenced by various factors including pH, amount and concentration of SCP, contact time, and magnetic condition time. The study observed varying adsorption rates for different metal ions under different conditions. Notably, the removal of ions by SCP increased with extended magnetic condition time and the addition of Fe_3_O_4_ nanoparticles. Both the Langmuir and Freundlich models effectively described the adsorption process for each ion adsorbed by SCP and magnetic SCP, with the Langmuir model generally showing a better fit across most treatments. This suggests that the Langmuir model more accurately describes the adsorption process for the studied ions on both SCP and Fe_3_O_4_‐SCP. Overall, the use of SCP and Fe_3_O_4_‐SCP for adsorbing Ni (II), Pb (II), Cu (II), and Fe (II) ions from desalinated water presents a promising and environmentally friendly wastewater treatment technique.

## 4 Experimental Section

### Adsorbent Preparation—*Sepia pharaonis* Cuttlebone Powder (SCP) Preparation

First, the cuttlefish bone of *Sepia pharaonis* was cleaned in three stages: washing with normal water, followed by a 5% sodium chloride solution, and finally with distilled water. After drying at room temperature in the laboratory, the inner oyster was cut into pieces and placed in an oven at 60 °C for 5 h. Once dried, the oyster pieces were powdered and sieved using a 100 µm mesh.

### Adsorbent Preparation—SCP‐Fe_3_O_4_ Preparation

0.125 grams of Fe_3_O_4_ sample, which had been synthesized previously, was dispersed in 0.5 mL of oleic acid (OA) and combined with 0.25 grams of SCP in a 50 mL solution of 2.0 wt.% acetic acid.^[^ ^43^ ^]^ This mixture was then placed in a beaker and subjected to stirring using an ultrasonic bath for a duration of 20 min. The presence of the acidic medium enhances the solubility of SCP and promotes its interaction with the Fe_3_O_4_ powder by protonating the amino groups found in the chitosan. The resulting mixtures were transferred to a glass reactor, stirred for an additional 20 min, and heated to 40 °C. To initiate a crosslinking reaction, 1 mL of glutaraldehyde (GA) was introduced to the prepared solutions. The aldehyde groups from GA then reacted with the amine groups of SCP powder, resulting in the formation of crosslinked polymer chains. This process was allowed to proceed for a total of 3 h. Subsequently, grey‐colored powders were separated from the liquid using magnetic decantation and washed multiple times with a mixture of deionized water and ethanol. Finally, the powders were dried in an oven at 50 °C.

### Characterization of Adsorbent

The adsorbent's surface functional groups were analyzed using Fourier transform infrared spectroscopy (FTIR) in the wave number range of 400 to 4000 cm^−1^, using a Bruker Instruments, Billerica, USA. Crystalline structure was determined using X‐ray diffraction (XRD) with a Siemens D 5000 diffractometer (Bruker, Germany), employing Cu Kα radiation (1.5418 Å) at a scan rate of 10° ^−1^min. SEM (VEGAA II, Czech) was employed for the surface evaluation of SCP. SEM images were obtained at 500 and 5000× magnifications.

### Adsorption Experiments

This study investigated the biosorption of nickel (II), lead(II), copper(II), and iron(II) ions using aqueous solutions.^[^ ^1^ ^]^ The experiment involved preparing 50 mL solutions with known metal ion concentrations, adjusting pH, and mixing them with varying amounts of adsorbent powder at 25 °C and 250 rpm for 10 min. After filtration, the remaining metal ion concentration was measured using a Shimadzu AA‐7000 flame atomic absorption device. To determine the concentration, a standard curve was created for each metal ion, plotting absorbance values against known concentrations within their linear ranges at specific wavelengths (283.3 nm for lead, 228.8 nm for iron, 295.6 nm for nickel, and 324.8 nm for copper). The concentration of metal ions in the filtered solutions was then determined by comparing their absorbance values to the corresponding standard curves.

### The Effect of pH on Adsorption

To optimize the pH of the adsorbent (SCP) for the biological removal of the mentioned heavy metals, separate solutions of each cation were prepared with a concentration of 20 mg L^−1^.^[^ ^38^ ^]^ These solutions were then mixed with 0.5 mg g^−1^ of SCP at pH levels ranging from 10 to 3. The mixtures were stirred at a speed of 250 rpm for a duration of 10 min at room temperature. The percentage of bioadsorption for each cation at different pH levels was calculated, and a corresponding curve was plotted to visualize the relationship between pH and the efficiency of bioadsorption for each metal.

### Effect of Time on Adsorption

To study the effect of contact time on the biosorption of SCP, individual solutions of each cation were prepared with a concentration of 20 mg L^−1^, while maintaining a constant pH.^[^ ^38^ ^]^ These solutions were then mixed with 0.5 mg g^−1^ of SCP and stirred at 250 rpm for a range of contact times, varying from 2 to 120 min at room temperature. Afterward, the solutions were filtered using filter paper, and the concentration of the desired cations was determined using an atomic adsorption device. Based on the obtained concentration values, the percentage of bioadsorption for each cation was calculated at different time intervals. A corresponding curve was then plotted to illustrate the relationship between contact time and the efficiency of bioadsorption for each metal.

### Effect of Magnetization Time on Adsorption

To investigate the effect of SCP magnetization time (1–400 min) on its bioadsorption capacity, separate solutions of each cation were prepared with specific concentrations and pH levels. The resulting solutions, each containing 0.5 mg g^−1^ of SCP, were stirred at 250 rpm at room temperature. Then, the solutions were filtered using filter paper, and the concentration of the desired cations was determined using an atomic adsorption device. Based on the obtained concentration values, the percentage of bioadsorption for each cation was calculated at different time intervals. Subsequently, a corresponding curve was plotted to illustrate the relationship between SCP magnetization time and the efficiency of bioadsorption for each ion.

### Fitting of Adsorption Isotherms

To explore the adsorption mechanism, the obtained experimental data was used to deduce adsorption isotherms. In this study, two commonly used models, namely the Langmuir (Equation 1) and Freundlich (Equation 2) models, were employed to establish the relationship between equilibrium adsorption capacity and equilibrium concentrations.^[^ ^1^ ^]^

(1) $$\frac{Ce}{Cq}=\frac{1}{Kl\times q\;\max}+\frac{Ce}{q\;\max}$$

(2) $$\ln\;q_{e}=\ln\;K_{f}+\left(1/n\right)\;\ln\;C_{e}$$

In the Langmuir model, it was assumed that the adsorbent surface was homogeneous and consists of only one type of binding site. On the other hand, the Freundlich model considers multi‐layer adsorption on a heterogeneous surface. By fitting the experimental data to these isotherm models, valuable insights can be gained into the adsorption behavior and mechanism of the studied system. Also, one of the important characteristics in the Langmuir isotherm that can indicate favorable surface adsorption was the separation factor RL, which was obtained by Equation (3).

(3) $$R_{L}=1/1+bC_{0}$$

## Conflict of Interest

The authors declare no conflict of interest.

## Data Availability

The data that support the findings of this study are available from the corresponding author, upon reasonable request.
